# Case Report: Extensive Dermatitis Secondary to Severe Malnutrition, Zinc and Vitamin Deficiencies After Malabsorptive Bariatric Surgery

**DOI:** 10.3389/fendo.2021.623543

**Published:** 2021-05-12

**Authors:** Aura D. Herrera-Martínez, Sonia Junquera-Bañares, Lucía Turrión-Merino, Francisco Arrieta-Blanco, José Botella-Carretero, Clotilde Vázquez-Martínez, Alfonso Calañas-Continente

**Affiliations:** ^1^ Endocrinology and Nutrition Service, Reina Sofía University Hospital, Córdoba, Spain; ^2^ Endocrinology and Nutrition Service, Ramón y Cajal University Hospital, Health Research Institute (IRyCIS), Madrid, Spain; ^3^ Dermatology Service, Ramón y Cajal University Hospital, Health Research Institute (IRyCIS), Madrid, Spain; ^4^ Endocrinology and Nutrition Service, Fundación Jimenez Diaz University Hospital, Madrid, Spain

**Keywords:** zinc deficiency, dermatitis, severe malnutrition, bariatric surgery complications, biliopancreatic diversion

## Abstract

Bariatric surgery is one of the most effective treatments currently available for obesity and its derived comorbidities. However, complications may occur, especially when malabsorptive surgeries like a biliopancreatic diversion is performed. We present the case of a female patient whose obesity was treated with this technique, and in the 9^th^ year of follow-up developed an extensive dermatitis secondary to zinc deficiency and malnutrition, precipitated by therapeutic non-compliance. A close surveillance of early symptoms and signs of nutritional deficiencies as well as chronic supplementation of vitamins and trace elements is required; this case illustrates the relevance of periodical, lifelong visits to a medical physician with special training and experience in the management of post bariatric surgery patients in order to prevent, diagnosis and early treat related complications.

## Introduction

Bariatric surgery is considered the most effective tool for long-term weight-loss maintenance in patients with severe obesity, it is also associated with significant reduction obesity-related comorbidities and mortality (1, 2). Biliopancreatic diversion (BPD) is a mixed bariatric surgery technique (restrictive and malabsorptive), it induces malabsorption due to reduced contact of ingested food with bile acids and pancreatic enzymes. BPD is less restrictive and more malabsorptive than the Roux-en-Y gastric bypass, it produces a sustained weight loss of 75 - 85% during 5 to 20 years of follow up (3, 4). The 50 centimeters common limb induces fat and polysaccharides malabsorption, reducing the main caloric intake obtained from these macronutrients. Specifically, several vitamin and mineral deficiencies are frequent despite vitamin supplementation, raising up to 80%; among them, iron deficiency, anemia, and fat-soluble vitamins deficiencies are frequently observed (5, 6). This is one of the most effective bariatric procedures to induce weight loss with long-term maintenance (3, 4), but due to the significant associated vitamin and nutients deficiencies, life-long monitoring of micronutrients at a specialized bariatric center are mandatory (6).

Bariatric surgery patients are usually malnourished before surgery despite an overconsumption of calories (7); several guidelines suggest to evaluate zinc and other vitamin/micronutrients levels before surgery, specially before mixed or malabsorptive procedures, since pre-surgery depletions are frequently observed (8–10). Importantly, patients with obesity have lower serum zinc in plasma and erythrocytes than leaner patients, probably due to increased urinary excretion because of hyperinsulinism (11); for this reason, specific repletion of zinc is indicated when signs and symptoms are evident and zinc assays are severely low (8).

BPD has potentially more risk of long-term nutritional complications than other techniques because of the extensive malabsorption induced by exclusion of a large portion of the stomach, the totality of the duodenum and a variable portion of the small intestine (5–7). Despite the use of vitamin supplementation, at least one nutritional deficiency is observed in up to 90% of BPD patients. Anemia (43%), hypocalcaemia, vitamin D (60%), A (28%), E (10%), K (60%) and zinc deficiency (33%) are frequently observed (5). Hypoalbuminemia is frequent affecting 25% of patients (5). Steatorrhea is responsible for several of these deficiencies, it results from the delayed and ineffective mix of the alimentary bolus with biliopancreatic secretions in the distal portion of the small intestine (common limb). For this reason, long-term supplementation and follow-up of blood plasma levels of liposoluble, hydrosoluble vitamins and different trace elements are necessary (7–10, 12, 13).

We present a patient with extensive dermatitis secondary to zinc deficiency and severe malnutrition after malabsorptive bariatric surgery.

## Case Report

A 42-year-old Caucasian woman underwent laparoscopic BPD in 2001. Her preoperative weight and body mass index (BMI) were 132.5 kilograms and 48.7 Kg/m^2^, respectively, with a body weight excess of 64 Kg (**Table 1**). Initially, the patient had appropriate follow-up and received vitamin supplementation during seven years until 2008, when lost to follow-up. Ten years after surgery (120 months), she came back to the outpatient clinic for follow-up, she was not receiving any vitamin supplement due to economic difficulties and work-related problems. In that moment, the patient had vitamin A, vitamin D, zinc, copper and iron deficiencies, as well as an elevated serum gamma-glutamyl transpeptidase (**Table 2**). Treatment was started with zinc sulphate (15 mg/day of elemental zinc), ferrous sulphate (40 mg/day), calcium carbonate and cholecalciferol (5,000 mg/day and 1760 IU/day, respectively), calcifediol (798 mcg/week), vitamin A (retinyl palmitate 50,000 IU daily), vitamin E (α-tocopherol acetate 200 mg/day), cyanocobalamin (1000 mcg/month) and two multivitamin supplements with minerals and trace elements. Education about diet and oral intake was reinforced.

**Table 1:** Anthropometric evaluation during the follow up.

Height: 1,65 m.	Weight (kg)	BMI (kg/m2)	Weight Loss (%)
Preoperative	132.5	48.7	
120 months (10-y) after surgery	86	31.6	- 46.5 kg 35.1%
150 months (11.5-y) after surgery	76.8	28.2	55.7 kg 42.0%
Hospital admission 153 months (12-y) after surgery	71.2	26.2	- 61.3 kg 46.3%
Hospital discharge 153 months (12-y) after surgery	74	27.2	- 58.5 kg 44.2%

BMI, body mass index.

**Table 2:** Serum laboratory results during hospital admission and follow up.

Parameter (reference range)	120-m (10-y) after surgery	150-m (11.5-y) after surgery	Hospital admission153-m (12-y) after surgery	Hospital discharge153-m (12-y) after surgery
Total serum protein (6.4-8.3 g/dl)	6.6	5.7	3.6	7.1
Albumin (3.3-5.2 g/dl)	4.3	3.3	**1.4 (L)**	3.4
Transferrine (200-360 ng/ml)	294	213	**93.2 (L)**	252
Retinol binding protein (3-6 mg/dl)	5.6	–	**1.3 (L)**	3.6
Prealbumin (20-40 mg/dl)	41.5	–	**10.8 (L)**	21.6
Aspartate aminotransferase (4-50 UI/L)	31	**60 (H)**	41	**101 (H)**
Alanine aminotransferase (5-40 UI/L)	23	**114 (H)**	36	**47 (H)**
Gamma-glutamyl transpeptidase (7-30 UI/L)	**164 (H)**	**927 (H)**	**346 (H)**	**990 (H)**
Alkaline phosphatase (42-141 UI/L)	88	130	130	**158 (H)**
Total bilirubin (0.2-1.2 mg/dl)	0.9	–	0.8	0.6
25-Hydroxyvitamin D (19.1-57.6 ng/ml)	**6.4 (L)**	23	**9.5 (L)**	23.1
Vitamin B_12_ (200-732 pg/ml)	266	_	**1349 (H)**	648
Zinc (60-150 µg/dl)	**49 (L)**	**42.3 (L)**	**44.4 (L)**	69
Copper (60-160 µg/dl)	**51 (L)**	_	**36 (L)**	77
Iron (60-150 µg/dl)	_	**22 (L)**	_	**45 (L)**
Ferritin (14-179 ng/ml)	**12.7 (L)**	43	21.2	20.1
Haemoglobin (12-18 g/dl)	12.9	**10.4 (L)**	**8.6 (L)**	**11.2 (L)**
Leukocytes (4-11 x 10^3^/µl)	8.1	5.9	5.6	7.6
Lymphocytes (1-3.5 x 10^3^/µl)	2.4	1.4	1.8	2.2
Vitamin A*	**18 (L)** (25-80 µg/dl)	22 (20-50 µg/dl)	**10.4 (L)** (30-60 µg/dl)	**29.2 (L)** (30-60 µg/dl)
Vitamin E*	613 (570-1670 µg/dl)	760 (500-2000 µg/ml)	**492.8 (L)** (500-1800 µg/dl)	708.9 (500-1800 µg/dl)

*Normal range for vitamins A and E are shown under every value due to different laboratory techniques. Abnormal values are highlighted. H, high; L, low.

One year later, 150 months after surgery, she was admitted in another hospital due to protein malnutrition, she presented with lower limb edema and a diffuse erythematous desquamating severe dermatitis over legs, abdomen and chest without ulcerations or oral lesions. Serum analysis showed hypozincemia, anemia, and elevated serum levels of aspartate aminotransferase, alanine aminotransferase and gamma glutamyl transferase (**Table 2**). Due to increased liver enzymes, a liver biopsy was performed and revealed severe steatohepatitis. Treatment was started with oral nutritional supplements (780 Kcal and 39 g protein/day), topical and systemic corticosteroids.

**Figure 1:**
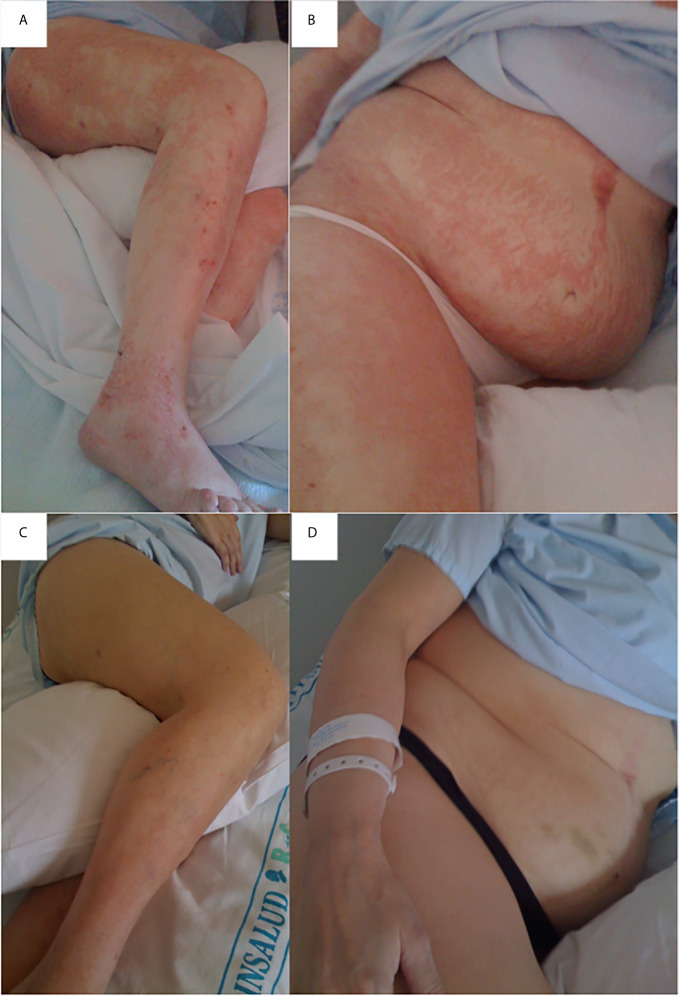
Erythematous desquamating dermatitis due to zinc deficiency. Lesions affect perioral, external genitalia and acral areas, with progressive dissemination to the rest of the body. Erythematous desquamating dermatitis affecting legs **(A)**, upper right leg, abdomen, chest and right arm **(B)**. Erythematous dermatitis in legs after three days of intravenous zinc supplementation and oral supplements administration **(C)**; improvement was also observed in the upper right leg, abdomen, chest and right arm **(D)**.

Twelve weeks later (153 months, 12 years after surgery), she consulted the endocrinology outpatient clinic due to increased skin lesion. She reported 1-year history of erythematous desquamating dermatitis, mainly perioral, in external genitalia and acral areas, with progressive dissemination to the rest of the body. In some areas, the lesions were initially blister-like, evolving to desquamative lesions with cutaneous xerosis. During the previous 12 months, she had lost 16 Kg weight. Additionally, 4-months prior to admission, she presented anorexia, diarrhea (15 to 20 depositions/day), severe asthenia, gait problems, lower limb edema and subjectively decreased urine output. At that moment, she was receiving ferrous sulphate (40 mg/day), cyanocobalamin (1000 mcg IM/month) and two multivitamin complexes; she was not taking calcium carbonate/citrate, vitamins A, E and D, or the zinc sulphate. Insisting on the importance of adherence to diet and vitamin supplements, treatment with zinc sulphate (30 mg/day), vitamin A (50,000 IU daily), calcifediol (798 mcg/week) and vitamin E (200 mg/day) was prescribed. Despite oral treatment with vitamin supplements, clinical symptoms worsened and two days later she was admitted into our hospital with the diagnosis of severe protein-energy malnutrition and extensive dermatitis twelve years after surgery (**Figures 1A, B**).

At hospital admission, physical examination showed a 53-year-old patient with regular general conditions, with lower extremity pitting edema, glossitis, angular cheilitis, extensive plaques with erythema and desquamation in her extremities, trunk, periorificial areas and genitalia, with diffuse alopecia and loss of axillary and pubic hair. BMI at that moment was 26.2 Kg/m^2^ (**Table 1**). Serum analysis showed anemia, decreased serum levels of vitamin A, E, D, zinc, copper, albumin, prealbumin, transferrin and retinol binding protein (**Table 2**). The hydrogen breath test confirmed bacterial overgrowth, the skin biopsy reported psoriasiform dermatitis with confluent parakeratosis and necrotic keratinocytes, lymphohistiocytic infiltrate and papillary dermal edema; these changes were compatible with carential dermatitis.

Intravenous zinc repletion was adminsitred during 72 hours (4 mg of sulphate heptahydrate zinc daily for 3 days in short IV push) along with oral treatment with pancrealipase supplements daily (54,000 U. Ph. Eur. of amylase; 75,000 U. Ph. Eur. of lipase and 3000 U. Ph. Eur. of proteases), vitamin A (50,000 IU/day), vitamin E (200 mcgs of tocoferol acetate/day), calcifediol (266 mcg twice a month), zinc sulphate (25 mg elemental zinc/day), copper (10 mg copper element daily) and a multivitamin supplement with minerals and trace elements. She also received intramuscular thiamine (100 mg/day for 7 days) and was started on bland, simple diet with an additional oligomonomeric, peptidic, glutamine-enriched, high-protein oral supplement (202 kilocalories/day; 21,1% protein; 13,7% fat; 65,2% carbohydrate). Additionally, a 7-day cycle of rifaximin (800 milligrams daily) for bacterial overgrowth was given.

Skin lesions started improving 48 hours after starting treatment in the hospital, progressive clinical recovery was observed resulting in normal bowel habit (2-3 stools/day) after 4 weeks of treatment, normalized several nutritional parameters, disappearance of edema and almost complete resolution of dermatitis (**Figures 1C, D**). Body weight at discharge was 74 Kg, with a BMI of 27.5 kg/m^2^ (**Table 1**). The patient presented with complete resolution of the skin lesions and normal laboratory values. Patient was discharged with oral treatment that included oral nutritional supplement, two multivitamin complexes, ferrous sulphate (40 mg/day), cyanocobalamin (1000 mcg IM/month) zinc sulphate (30 mg/day), vitamin A (50000 IU daily), calcifediol (798 mcg/week) and vitamin E (200 mg/day). Follow-up was performed one and three months after discharge, the patient was asymptomatic and had appropriate compliance of oral treatment. The patient moved to another city and referred for correct treatment supplementation and follow-up.

## Discussion

Protein malnutrition is a potential severe complication of bariatric surgery (13), it is secondary to surgery-complications or non-compliance with pharmacological/nutritional treatment, affecting especially patients with persistent diarrhea or vomiting (14). Its depends on the volume of the gastric reservoir, the diameter of the gastrojejunal stoma, the length of the alimentary limb (<200cm), the common limb (<100cm) and the presence of bacterial overgrowth (15). It is especially frequent in malabsortive procedures, including BPD, duodenal switch, one anastomosis gastric bypass and single anastomosis duodeno-ileal bypass with sleeve gastrectomy (SADIs) (5, 16); since deficits are observed even in 90% of patients, considerable negative side effects should not be underestimated (5). A retrospective analysis has described that a 150-cm length of the biliopancreatic limb is related with minimal nutritional complications, while a 250-cm limb is associated with significant nutritional deficiencies (17). Malnutrition even requires hospitalization and extension of the common channel in some patients (5).

Zinc is the second most abundant trace element in the human body after iron, it binds proteins in practically all types of cells. About 20 to 40% of dietary zinc is absorbed in the small intestine, mainly in duodenum and jejunum, and in lesser quantity in the ileum (18, 19). Hypozincemia is highly prevalent in patients with BPD, because the principal areas of zinc absorption are excluded (19). Zinc deficiency is characterized by dermatitis, diarrhea and alopecia, but only 20% of the patients have all three of these features at the moment of diagnosis.

Skin lesions are usually the earliest signs of hypozincemia (20). Patients often present with angular cheilitis, paronychia, alopecia, photophobia, and/or eczematous plaques that evolve into vesiculobullous and pustular lesions, skin cell apoptosis is the possible physiopathological mechanism for these clinical alterations (21). The severity of cutaneous lesions seems to be related with the magnitude and velocity of instauration of zinc depletion, which could explain differences in clinical presentation according different zinc deficiency levels (21, 22).

Atrophy of intestinal villi, chronic inflammation of the intestinal mucosa, bacterial infections or candidiasis (Candida albicans) are common and can also produce diarrhea (19, 23). In this patient, diarrhea could have been either a cause of hypozincemia (because of intestinal loss) or a symptom of the deficiency, in both cases, bacterial overgrowth worsened the clinical presentation.

Finally, anorexia due to zinc deficiency, presents also with smell-, taste-disorders, and subsequent weight loss (24). According to some studies, zinc might regulate serum leptin levels in humans and insulin secretion, playing an important role in appetite regulation (25). Despite alopecia is a very frequent symptom of zinc deficiency, zinc concentration in hair is an unreliable parameter of acute deficiency; hair growth can be slowed down or halted with normal zinc concentrations, thus zinc hair levels are only decreased on low-grade chronic deficiency (26).

Zinc deficiency after BPD has an incidence of 25-45% despite multivitamin supplements (15) but only few cases have significant clinical features. To the best of our knowledge, there are only five described cases of acrodermatitis due to zinc deficiency secondary to bariatric surgery (27). Importantly, liver injury was also observed in this patient. Specifically, steatohepatitis leading to liver cirrhosis following BPD is related to rapidly weight loss, bacterial overgrowth, macro-and micro-nutrients deficiencies and severe malnutrition (28), for this reason, liver enzymes are routinely monitored in bariatric surgery patients (29).

This patient presented with other significant vitamin and trace elements deficiencies, cupper, vitamin A and E, which may have also influenced the clinical presentation and evolution. When patients present with clinical symptoms and several deficits, all them should be corrected according following the clinical recommendations (6, 8). Regarding this, bariatric surgery is the most frequent cause of acquired copper deficiency. Copper is absorbed in the stomach and proximal duodenum; it is required for the production of red and white blood cells and is involved in the proper functioning of the nervous system (30). Copper deficiency results in hematological microcytic anemia, leukopenia) and neurological alterations affecting the posterior tracts of the spinal cord (similar to those produced by vitamin B12 deficiency), even optic and other cranial nerves. Vitamin E deficiency results in myopathies and neuropathies such as sensory axonopathies, chiefly involving the posterior column, nerve roots, and peripheral nerves. Deficiency of vitamin A is rarely symptomatic due to large liver reserves, but it produces xerophthalmia, night blindness, anemia, and skin alterations (8, 10).

Interestingly, despite severe malnutrition, this patient did not present with thiamin deficiency, which is associated with vomiting and rapid weight loss. Clinically it is presented as a peripheral polyneuropathy, also known as bariatric beriberi, neuritic or dry beriberi (8, 10). It is the most common cause of postoperative bariatric polyneuropathy. The disorder mainly affects the two lower limbs symmetrically and is of the axonal demyelinating type with mixed sensory and motor features. It may develop on its own or in association with Wernicke-Korsakov encephalopathy. Similarly to neurological manifestations due to vitamin B12 deficiency, improvement following thiamin replacement is variable and relatively slow (31).

Despite the skin lesions rapidly improved after zinc, vitamin and micronutrients supplementation, we do not consider a spontaneous recovery of a transient event in this patient, since the patient reported one-year history of erythematous desquamating dermatitis that was progressively impairing in parallel to increased weight loss and edema.

Lifelong monitoring following bariatric surgery is necessary to ensure that nutritional requirements are met, and post‐bariatric surgery–related nutritional deficiencies and complications are reduced. Time intervals and analysis varies according to the bariatric procedure and should be individualized. In general, close follow-up is recommended during the first two years, and then it should be performed annually (8, 10).

Despite BPD is not a common surgical procedure nowadays, this case reflects the importance of properly long term follow-up, monitoring, and nutritional therapy in order to prevent, early diagnose and/or treat various deficiency states (29, 32, 33).

## Data Availability Statement

The original contributions presented in the study are included in the article/supplementary material. Further inquiries can be directed to the corresponding authors.

## Ethics Statement

Ethical review and approval was not required for the study on human participants in accordance with the local legislation and institutional requirements. The patients/participants provided their written informed consent to participate in this study. Written informed consent was obtained from the individual(s) for the publication of any potentially identifiable images or data included in this article.

## Author Contributions

All authors have equally contributed to this article. All authors contributed to the article and approved the submitted version.

## Funding

Instituto de Sldu Carlos III JR19/00050. Nutricia kindly contributed with the publication fee of this article. The funder was not involved in the study design, collection, analysis, interpretation of data, the writing of the manuscript or the decision to submit it for publication.

## Conflict of Interest

The authors declare that the research was conducted in the absence of any commercial or financial relationships that could be construed as a potential conflict of interest.
